# Adipocyte fatty acid-binding protein (FABP4) as a potential biomarker for predicting metabolically driven low-grade and organ damage in thalassemia syndromes

**DOI:** 10.1007/s00277-024-05886-7

**Published:** 2024-07-19

**Authors:** Eman Mahmoud Ezzat, Salwa Bakr, Rehab M. Golam, Basma Atiya Abdelgyed, Nourhan Mohamed Nasr

**Affiliations:** 1 Department of Internal Medicine, Faculty of Medicine, Fayoum University, Fayoum, Egypt; 2 Department of Clinical Pathology/Hematology & Transfusion Medicine, Faculty of Medicine, Fayoum University, P.O. Box: 63514, Fayoum, Egypt; 3 Department of Medical Biochemistry and Molecular Biology, Faculty of Medicine, Fayoum University, Fayoum, Egypt

**Keywords:** Thalassemia, FABP4, Cardiometabolic metaflammation

## Abstract

Adipocyte fatty acid-binding protein (A-FABP; FABP4) plays a significant role in the pathogenesis and progression of metabolically driven low-grade inflammation and organ damage. This study aimed to evaluate the performance of circulating FABP4 as a predictive and diagnostic biomarker for thalassemia-associated cardiometabolic events. This case-control study enrolled 50 adults with β-thalassemia and 30 age-, sex-, and body mass index-matched controls. Participants underwent a comprehensive evaluation, including complete blood count, liver and kidney function tests, serum blood glucose, lipid profile, and ferritin levels, pelviabdominal ultrasound, ECG, and echocardiography after taking a full medical history and conducting a clinical examination. Serum levels of FABP4 were measured using an Enzyme-Linked-Immunosorbent-Assay. The diagnostic performance of FABP4 was assessed using receiver operator characteristic (ROC) curve analysis to determine optimal values for excluding and confirming cardiometabolic metflammation. The thalassemia cohort exhibited a statistically significant higher concentration of FABP4 compared to the control group (*p*-value < 0.001). Positive correlations were found between FABP4 and ferritin serum levels above 800 or 1000 ug/L, as well as with ALT, TGS, and LDL (*p*-value < 0.05). Circulating FABP4 was identified as a statistically significant risk factor for thalassemia-associated cardiometabolic comorbidities (OR = 84.00, 95%CI:18.6–378.6, *p*-value < 0.001). ROC analysis determined that the FABP4 exclusionary cut-off value > 2.30 ng/ml could effectively discriminate between thalassemia-associated adverse metaflammation and controls, while the FABP4 confirmatory cut-off value was > 2.58 ng/ml. In conclusion, circulating FABP4 appears to be a potential risk factor for predicting progression to cardiometabolic events in thalassemia-associated adverse metaflammation. FABP4 holds promise as a diagnostic and prognostic biomarker for disease monitoring and risk stratification. Further validation through large-scale, multicenter, prospective studies is warranted.

## Introduction

Thalassemia syndrome is an inherited erythrocyte disorders related to hemoglobin synthesis, classified under hemoglobinopathies, and constitutes a significant global health burden [1]. In β-thalassemia, clinical severity varies widely, from asymptomatic carriers to severe or even fatal forms, depending on the degree of imbalanced production of globin chains (α and β) [2].

Cardiometabolic comorbidities, including various endocrinopathies such as diabetes mellitus, hypogonadism, parathyroid and thyroid dysfunction, osteoporosis, gallbladder and renal lithiasis, and hepatic harmful consequences, are among the most common issues in thalassemia patients. These complications arise due to risks associated with iron overload, oxidative stress, chronic inflammation, and tissue damage [3]. Severe forms of thalassemia are characterized by severe anemia and regular transfusion dependency (TDT) every 2–6 weeks for survival, which increases the risk of excessive iron overload [1, 4]. Non-transfusion-dependent thalassemia (NTDT) patients, who may require occasional red blood transfusions in certain situations like pregnancy, surgery, or infection, are also at risk of iron overload due to enhanced iron absorption [5].

Significant evidence links higher serum ferritin levels (above 1000 µg/L) with iron overload due to chronic transfusion and increased morbidity and mortality, especially if untreated [6]. However, studies have also shown that ferritin serum levels above 800 ug/L are associated with clinical morbidity in NTDT [5]. Consequently, in 2021, the British Society of Haematology (BSH) updated its guidelines for monitoring iron overload and managing thalassemia patients receiving regular transfusions every three months or less (TDT) [7].

Fatty acid-binding proteins (FABPs) are small, soluble, intracellular lipid-binding protein family (14–15 kDa) essential for modulating free fatty acid fluxes transporting them to various cellular compartments and specific enzymes (e.g., lipid droplets for storage, endoplasmic reticulum for re-esterification, mitochondria and peroxisomes for oxidation, or nucleus for gene regulation). They play role in solubility, lipid and glucose metabolism, and energy storage [4]. This family includes tissues highly active in fatty acid metabolism, such as adipocyte (A-), brain (B-), heart (H-), liver (L-), epidermal (E-), myelin (M-), testis (T-) intestinal (I-), and ileal (Il-) FABPs [8].

Adipocyte FABP (A-FABP), also known as FABP4 or adipocyte protein 2 (aP2), is a secreted adipokine hormone abundantly expressed in adipose tissue and, to a lesser extent, in macrophages upon differentiation from monocytes [9]. It functions as a lipid chaperone protein that regulates various aspects of metabolic and inflammatory immune response pathways [4]. Although circulating FABP4 plays different physiological regulatory roles in responding to life-threatening circumstances, such as maintaining glucose homeostasis or enhancing communication between energy-storage systems and remote target organs [10], its chronic persistent engagement under distinct conditions of immunometabolic stress terminated with uncontrolled lipolysis; such as obesity (genetic /diet-induced), starvation, insulin resistance, or hypercholesterolemia exacerbates numerous immunometabolic disorders, including atherosclerosis, diabetes mellitus, hypertension, acute ischemic stroke, cancer, and asthma [11, 12]. Overall, atypical inflammation emerging from metabolic tissues is termed metaflammation [10].

Previous studies have shown that FABP4 concentration in blood is associated with cardiovascular and metabolic abnormalities [11, 13]. As a result, FABP4 has been identified as a common risk factor and biomarker for adiposity and metaflammation in metabolic syndrome [8, 9]. Although various adipokines have been implicated in the progression of thalassemia complications, the correlation between FABP4 levels in thalassemia and the development of cardiometabolic comorbidities is not well understood. Given the chronic inflammation observed in thalassemia, FABP4 levels may reflect the inflammatory burden and predict cardiovascular risk. Additionally, the role of FABP4 in lipid metabolism and oxidative stress makes it a relevant marker for monitoring lipid-related complications and could potentially indicate the extent of cardiac injury [11].

Therefore, FABP4 levels could serve as an early marker for subclinical cardiometabolic disturbances in thalassemia patients. This study aimed to evaluate the performance of circulating FABP4 as a predictive and diagnostic biomarker for thalassemia-associated cardiometabolic events. Understanding the correlation between circulating FABP4 and metaflammation-associated risks in thalassemia could highlight its potential as a therapeutic target for preventing or treating immunometabolic comorbidities in these patients [11].

## Materials and methods

### Study design and sample population

This case-control study enrolled 80 adult participants, including 50 thalassemia patients from the outpatient clinic of Fayoum University Hospital, and 30 age-, sex-, and body mass index (BMI)- matched controls. Obese individuals with a BMI of 30 or above, as defined by the WHO, were excluded. The study adhered to the Helsinki ethical standards after obtaining approval from the institution Ethical Committee.

A comprehensive history was obtained for each participant, including the frequency of blood transfusions and iron chelation, recent infections, and clinical examination. Written informed consent was obtained prior to the collection of peripheral blood samples. Each participant underwent a thorough evaluation, including complete blood count, liver and kidney function tests, lipid profile, fasting blood glucose (FBG), serum ferritin, ESR, high sensitivity C-reactive protein (Hs-CRP), pelviabdominal ultrasound, electrocardiography (ECG) and echocardiography (Echo). Thalassemia patients were further classified based on transfusion frequency into those with TDT and NTDT.

To ensure accurate identification and diagnosis of cardiometabolic comorbidity, precise diagnostic criteria were employed. Cardiomyopathy was diagnosed based on clinical criteria and ECG, with Echo evidence of reduced Left Ventricular Ejection Fraction (LVEF) < 55% indicating systolic dysfunction. Heart Failure was defined based on clinical criteria and imaging studies, with symptoms including shortness of breath, fatigue, and edema. Echo evidence of reduced LVEF (< 55%) indicated systolic dysfunction, supported by elevated B-type natriuretic peptide (BNP) levels, typically > 100 pg/mL. Pulmonary hypertension was defined by elevated mean pulmonary arterial pressure (mPAP ≥ 25 mmHg at rest), measured by right heart catheterization, while Echo estimates of systolic pulmonary arterial pressure > 35 mmHg were used for screening. Arrhythmias were identified through ECG or Holter monitoring, with findings including atrial fibrillation, atrial flutter, ventricular tachycardia, or other significant rhythm abnormalities. Holter monitoring detected frequent premature ventricular contractions, runs of non-sustained ventricular tachycardia, or other arrhythmias. Hypertension was defined by blood pressure measurements of systolic blood pressure (SBP) ≥ 130 mmHg and diastolic blood pressure (DBP) ≥ 80 mmHg, or on treatment with anti-hypertensive medication. Ambulatory Blood Pressure Monitoring confirmed hypertension in borderline cases.

For diagnosing of common metabolic disorders, dyslipidemia was defined based on serum lipid profile levels: elevated total cholesterol ≥ 200 mg/dL, Low-Density Lipoprotein Cholesterol (LDL-C) ≥ 130 mg/dL, reduced High-Density Lipoprotein Cholesterol (HDL-C) < 40 mg/dL for men and < 50 mg/dL for women, and elevated triglycerides ≥ 150 mg/dL). Delayed puberty and short stature were assessed through physical examinations, growth measurements, bone age assessments, and hormonal evaluations. Diabetes Mellitus (DM) was diagnosed based on fasting blood glucose levels ≥ 126 mg/dL on at least two occasions and/ or Hemoglobin A1c (HbA1c) level ≥ 6.5%, or Oral Glucose Tolerance Test (OGTT) 2-hour plasma glucose levels ≥ 200 mg/dL.

### Laboratory testing

Enzyme-Linked Immunosorbent Assay (ELISA) was performed using the ChroMate 4300 (Awareness Technology, Inc., USA) to measure serum levels of FABP4 for all participants, using a kit from Bioassay Technology Laboratory (BT Lab, Shanghai, Chain) and following the manufacturer’s instructions.

### Statistical analysis

Data were analyzed using Statistical Package for Social Science software version 22 (SPSS Inc., Chicago, IL, USA). Descriptive statistics were presented as numbers and percentages for qualitative data, and arithmetic means and standard deviations for quantitative parametric data. Quantitative data were first tested for normality using the One-Sample Kolmogorov-Smirnov test in each study group, followed by appropriate inferential statistic tests. The Chi-square test was used for comparisons between groups. Quantitative data were expressed as range, mean, standard deviation, and median. The Student’s t-test or Mann-Whitney test was applied to compare two groups based on the distribution of variables. The Bivariate Spearman Coefficient was used for correlation estimation. Receiver Operating Characteristic (ROC) curve analysis assessed the sensitivity and specificity of the new test. A *p*-value < 0.05 was considered statistically significant.

## Results

The present study enrolled 50 thalassemia patients with a mean age of 28.7 ± 8.61 years, including 54% with thalassemia major and 46% with thalassemia intermedia, as well as 30 age-, sex-, and BMI-matched healthy individuals. According to the WHO definition, 47 patients were non-obese and had average waist circumference, while three were overweight. The sociodemographic and clinical data are detailed in Table (1).

**Table 1:** Comparison between the two studied groups according to demographic, clinical, Investigation Data and FABP4 serum level

	Thalassemia Patients (*n* = 50)	Control (*n* = 30)	*P*-value
**Mean ± SD; Median (Min – Max)**			
**Age** *(years)*	28.6 ± 8.5	24.4 ± 4.2	0.055
**BMI***(Kg/m*^*2*^)	22.5 ± 3.47	22.21 ± 2.47	0.450
**Blood Pressure (SBP/ DBP)** mmHg	119 ± 16.36 / 82.53 ± 7.20	117.01 ± 6.05 / 83 ± 4.86	0.25 / 0.40
**No. (%)**			
**Gender**: Male	26 (52%)	15 (50%)	1
Female	24 (48%)	15 (50%)	
**History of Previous Transfusion**:	42 (86%)	0 (0%)	<0.001*
**Splenectomy**	28 (56%)	0 (0%)	< 0.001*
**Cholecystectomy**	2 (4%)	0 (0%)	0.5
**Iron Chelator Current Administration**	35 (70%)	0 (0%)	<0.001*
**Laboratory Investigations: Mean ± SD; Median (Min – Max)**			
ALT (IU/L)	38 (7-2.03)	35 (15–56)	0.1
AST (IU/L)	41.5 (18–217)	32 (15–56)	0.002*
Total bilirubin (mg/dL)	1.7 (0.44–6.5)	0.96 (0.74–1.8)	0.001*
Direct bilirubin	0.41(0–20)	0.33 (0–1)	0.001*
Albumin	4.1 ± 0.34	4.3 ± 0.34	0.04*
Hemoglobin (g/L)	8.01 ± 1.2	13.8 ± 1.1	< 0.001*
Platelet	463.6 ± 197.6	309.3 ± 77	< 0.001*
TLC	9.1 ± 3.9	7.04 ± 2.4	0.009*
Ferritin (ug/L)	1450 (300–5000)	43.5 (9–98)	< 0.001*
Total Cholesterol (mg/dL)	132.7 ± 31.9	130.03 ± 26.3	0.7
TGS (mg/dL)	149.4 ± 48.9	108.7 ± 18.6	< 0.001*
LDL (mg/dL)	73.5 ± 27.2	86.2 ± 9.1	0.01*
HDL (mg/dL)	36.5 ± 8.2	44.3 ± 6.7	< 0.001*
FBG (mg/dL)	94.9 ± 35.6	92.1 ± 8.3	0.7
2HPP- BG (mg/dL)	129.5 ± 44.9	--	----
ESR	15 (4–70)	10 (5–22)	0.003*
CRP (mg/L)	6 (0–60)	1 (0–8)	< 0.001*
Urea	22.2 ± 5.9	27.7 ± 6.5	< 0.001*
Creatinine	0.53 ± 0.16	0.64 ± 0.15	0.005*
**Echo findings**			
LA	3.5 (3-4.9)	2.6 (2-3.2)	< 0.001*
LVED	4.9 (3.6–6.2)	3.8 (3.8–38)	< 0.001*
LVES	3.2 ± 0.43	2.4 ± 0.11	< 0.001*
PASP	29.6 ± 10.6	19 ± 1.6	< 0.001*
EF	63.9 ± 4.9	67.7 ± 2.2	< 0.001*
FS	34.3 ± 5.9	38.1 ± 0.99	0.001*
**TR No. (%)**			
- Normal	19 (38%)	30 (100%)	< 0.001*
- Mild	16 (32%)	0 (0%)	
- Moderate	13 (26%)	0 (0%)	
- Sever	2 (4%)	0 (0%)	
**ECG**			
- Normal	37 (74%)	30 (100%)	0.02*
- Sinus Tachycardia	11 (22%)	0 (0%)	
- AF	1 (2%)	0 (0%)	
- LT BBB	1 (2%)	0 (0%)	
**FABP4 serum level**	2.9 (1.2–17.3)	2.1 (1.4–2.3)	< 0.001*

* Statistically significant at *p*-value < 0.05

The majority of patients (72%) were on regular monthly blood transfusions (TDT), with an annual transfusion duration ranging from 0 to 46 months (median 20.50 months) and an annual chelation duration ranging from 0 to 39 months (median 12.00 months). Among these TDT patients, 75% had thalassemia major, and 25% had thalassemia intermediate. They exhibited statistically higher levels of serum ALT, total bilirubin, ESR, and ferritin (*p*-values < 0.05).

Data analysis also revealed that a significant proportion of patients (64%) had multiple endocrinopathies and metabolic comorbidities, with high incidences of dyslipidemia, gall bladder stones, and short stature/ delayed puberty (52%, 38%, and 33.8% respectively). Additionally, 50% of the patients had various subclinical cardiac problems, with high incidences of pulmonary hypertension and tricuspid regurgitation (44%, and 30% respectively).

Comparison with control groups showed significantly higher serum FABP4 levels (*p*-value < 0.001) among thalassemia patients. Significant differences were also observed between the two groups regarding echo findings and numerous laboratory investigations (*p*-values < 0.05, Table 1).

The study results revealed a significant positive correlation (*p*-value < 0.05) between serum FABP4 and levels of ALT, TGS, LDL, and ferritin. However, there was no significant correlation between FABP4 concentration and Echo findings (Table 2).

**Table 2:** Correlation between serum FABP4 level and thalassemia patient’s routine investigations:

Variables	FABP4	
	*r*	*P*-value
**Laboratory Investigations**:		
**ALT**	0.33	0.02*
AST	0.26	0.07
Total bilirubin	0.08	0.60
Direct bilirubin	-0.11	0.46
Albumin	0.09	0.54
FBS	-0.2	0.91
2HPP	0.02	0.88
ESR	0.17	0.24
CRP	-0.005	0.97
Cholesterol	0.24	0.08
**TGS**	0.44	0.001*
**LDL**	0.32	0.02*
HDL	-0.09	0.54
Urea	-0.21	0.14
**Creatinine**	-0.28	0.04*
**Ferritin**	0.38	0.007*
**Echo Finding**:		
LA	-0.23	0.84
LVED	0.14	0.31
LVES	0.09	0.53
PASP	0.19	0.18
EF	-0.13	0.36
FS	-0.15	0.28

*Statistically significant at *p* ≤ 0.05

Additionally, significantly higher levels of serum FABP4 were found among patients with ferritin levels above 800 and 1000 ug/L (*p*-value < 0.001, Table 3). However, no significant correlation was observed among the few patients (10 out of 50) with ferritin levels above 2500 ug/L (*p*-value 0.451). Additionally, there was no significant correlation between the duration of transfusion (*p*-value 0.23) or the duration of chelation (*p*-value 0.35) and FABP4 levels.

**Table 3:** Relation between circulating FABP4 concentration and serum ferritin levels in Thalassemia Cohort:

Serum Ferritin	FABP4 Median (Min – Max)	U	*p*-value
**≤ 800** (*n* = 6)	2.1 (1.4–4.6)	-5.6	< 0.001*
**> 800** (*n* = 44)	3 (1.2–17.3)		
**≤ 1000** (*n* = 15)	2.2 (1.4–4.6)	-4.9	<0.001*
**> 1000** (*n* = 35)	3.1 (1.2–17.3)		

*Statistically significant at *p* ≤ 0.05. U: Mann Whitney test

An interesting finding from the logistic regression analysis showed that FABP4 concentration was a statistically significant risk factor for overall cardiometabolic comorbidities (OR = 84.00, 95%, CI: 18.6–378.6, *p*-value < 0.001, Table 4).

**Table 4:** Univariate Analysis of FABP4 risk factor for Cardiometabolic complications in Thalassemia patients

Thalassemia-associated Cardiometabolic Comorbidities	Serum FABP4		
	OR	95%CI of OR	*P*-value
Metabolic complication	2.48	0.47–12.9	0.35
Cardiac complication	2.66	0.75–9.45	0.22
Endocrine complication	95.00	10.2-882.04	< 0.001*
Overall Cardiometabolic**	84.00	18.6-378.6	< 0.001*

*Statistically significant at *p* ≤ 0.05

**Cardiometabolic including endocrinopathies

**Fig. 1:**
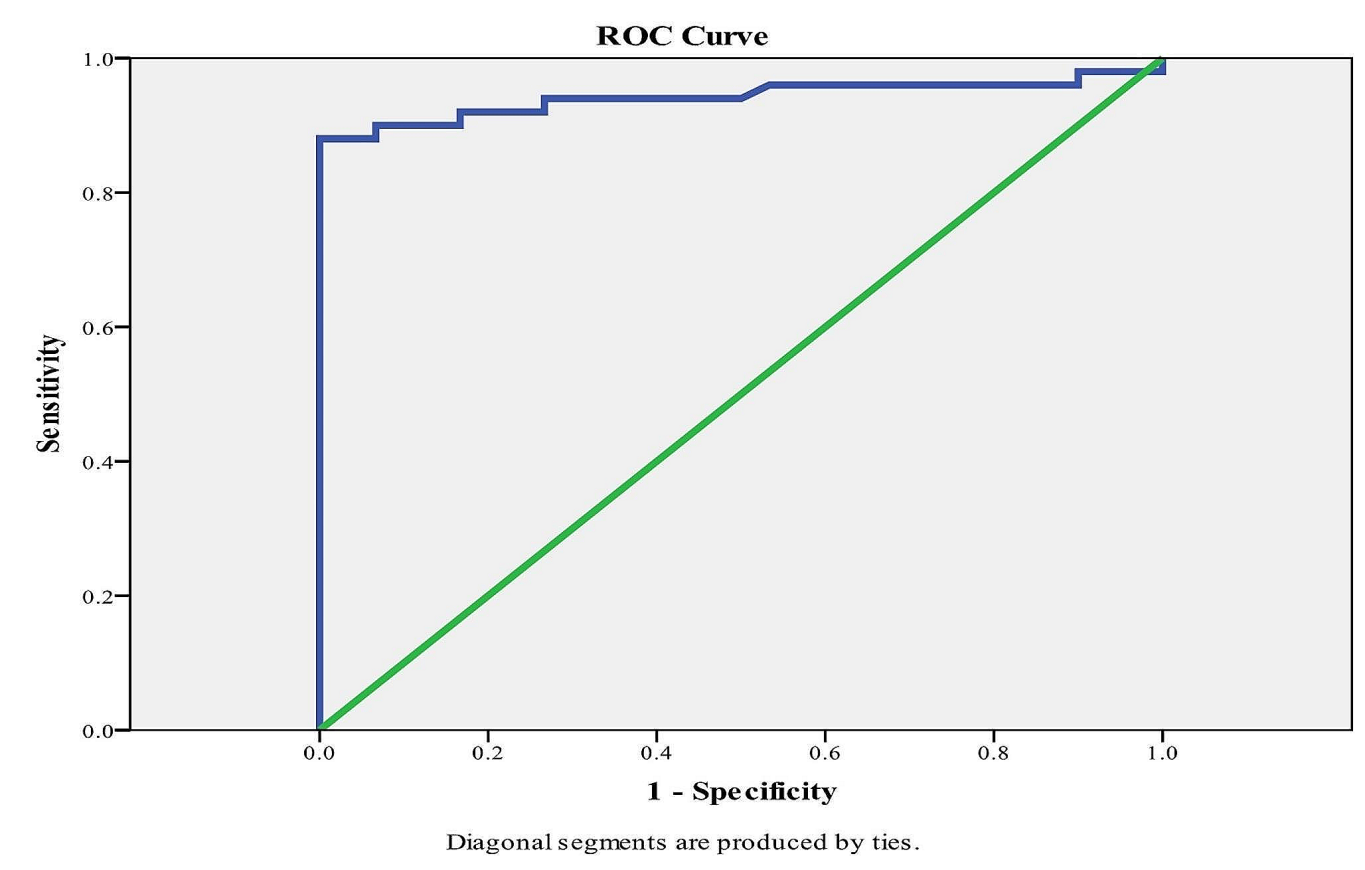
ROC Curve for FABP4 Based Diagnosis to Exclude “Rule out” Thalassemia-associated Cardiometabolic Events

ROC analysis demonstrated that a FABP4 value > 2.30 ng/ml was the exclusionary cut-off point to differentiate and rule out thalassemia-associated cardiometabolic/ adverse metaflammation cases from non-obese controls (Table 5), with 90% sensitivity and 93.3% specificity (Fig. 1).

**Table 5:** Predictive performance for FABP4 for discriminating patient metaflammation from non-obese control

	AUC	*p*-value	95% C. I	Cutoff	Specificity	Sensitivity	NPV	PPV
**FABP4**	0.942	< 0.001*	0.885–0.998	> 2.30**	93.3%	90%	84.8%	95.7%

*Statistically significant at *p* ≤ 0.05

**Normal cutoff value, AUC: Area under a curve; *P*-value: Probability value; CI: Confidence intervals; NPV: Negative predictive value; PPV: Positive predictive value

**Fig. 2:**
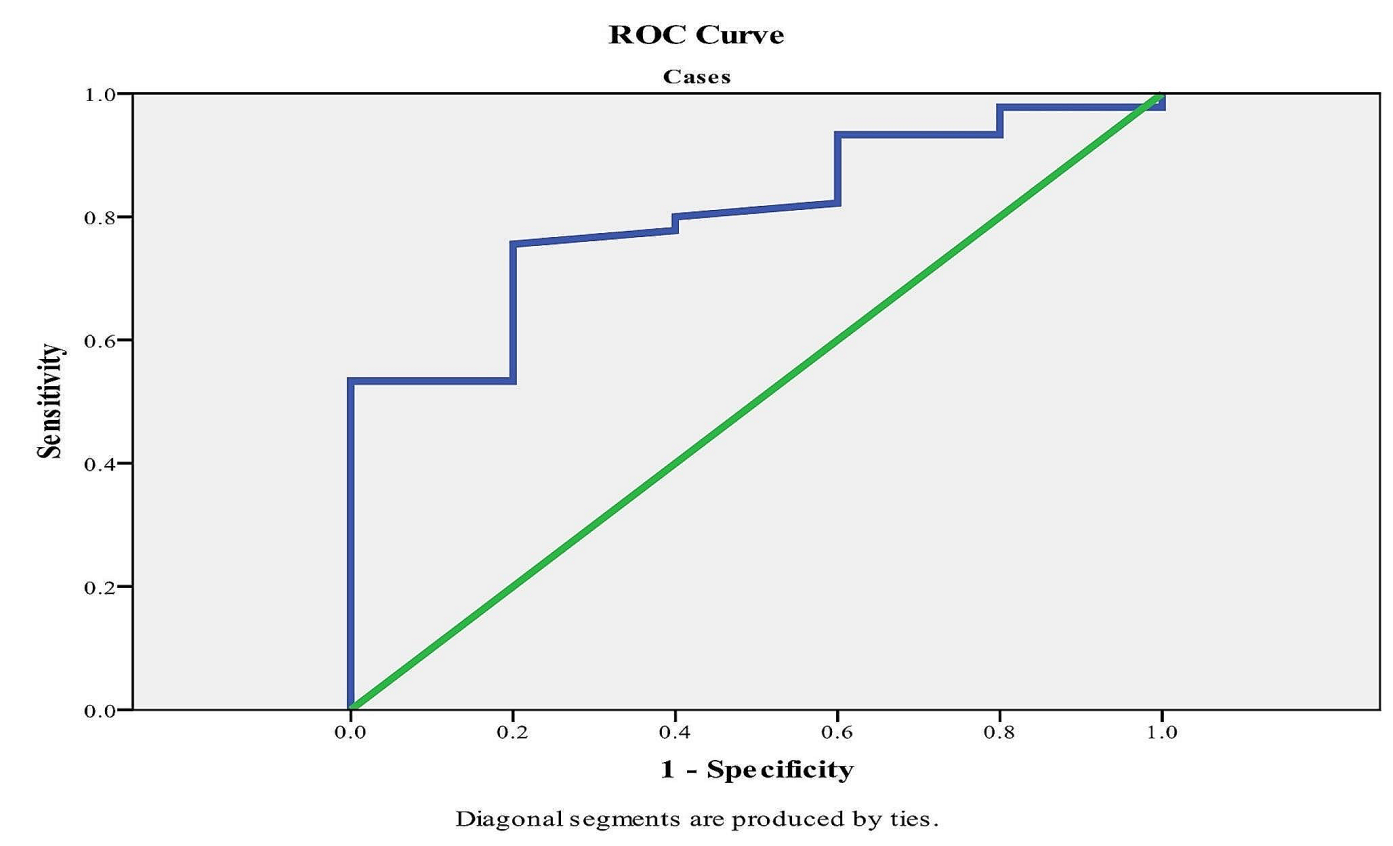
ROC Curve for FABP4 Based Diagnosis to Confirm “Rule in” Thalassemia-associated Cardiometabolic Comorbidities

Further, circulating FABP4 showed a significant diagnostic effect on overall thalassemia-associated cardiometabolic complications (*p*-value < 0.02, Table 6). The diagnostic accuracy of FABP4 for cardiometabolic complications revealed that an FABP4 confirmatory cut-off value > 2.58 ng/ml could rule in cardiometabolic comorbidities with 80% sensitivity and 60% specificity (Fig. 2). The diagnostic performance of FABP4 for overall cardiometabolic comorbidity is detailed in Table 6.

**Table 6:** Diagnostic performance for FABP4 for Cardiometabolic comorbidities in Thalassemia patients:

FABP4	AUC	*p*-value	95% C. I	Cutoff	Specificity	Sensitivity	NPV	PPV
**Overall Cardiometabolic**	0.804	0.02*	0.643–0.965	2.58***	60%	80%	94.7%	25%
**Metabolic**	0.877	< 0.001*	79.8-0.956	2.44	76.7%	89.2%	89.2%	76.7%
**Endocrine**	0.967	< 0.001*	0.933-1	2.94	91.7%	95%	98.2%	79.2%
**Cardiac**	0.863	< 0.001*	0.779–0.947	2.68	72.7%	80%	88.9%	57.1%

*Significant value at *p* ≤ 0.05

**Risk Cut-off Value for cardiometabolic complications, AUC: Area under a curve; *P*-value: Probability value; CI: Confidence intervals; NPV: Negative predictive value; PPV: Positive predictive value

Furthermore, highly significant differences were observed between the exclusionary FABP4 cut-off value (> 2.30) and thalassemia-associated cardiometabolic adverse events (*p*-value ≤ 0.05, Table 7).

**Table 7:** Comparison between FABP4 exclusionary cutoff value (> 2.3 ng/ml) for Predicting Thalassemia-Associated adverse metaflammation of cardiometabolic events in Study Population

Variables	No. (%) (*n* = 80)	FABP4		χ2	*P*-value
		Normal (≤ 2.3) (*n* = 33)	High (> 2.3) (*n* = 47)		
Metabolic disorders:					
- Gallstones	18 (22.5%)	2 (6.1%)	16 (61.7%)	9.1	0.01*
- Cholecystectomy	2 (2.5%)	0 (0%)	2 (4.3%)	1.4	0.6
- Dyslipidemia	26 (32.5%)	2 (6.1%)	24 (51.1%)	17.9	0.001*
- Leg ulcer	1 (1.3%)	0 (0%)	1 (2.1%)	0.7	1
**Endocrine complication**:					
- Diabetes mellitus	1 (1.3%)	0 (0%)	1 (2.1%)	1.4	0.5
- Short stature	10 (12.5%)	0 (0%)	10 (21.3%)	8.02	0.004*
- Delayed Puberty	17 (21.3%)	0 (0%)	**17 (36.2%)**	15.2	< 0.001*
- Hyperparathyroidism	1 (1.3%)	0 (0%)	1 (2.1%)	0.7	1
**Cardiac Complication**:					
- Chronic heart failure	5 (6.3%)	0 (0%)	5 (10.6%)	3.7	0.07
- Pulmonary hypertension	22 (27.5%)	1 (3%)	21 (44.7%)	16.9	< 0.001*
- Arrythmias and heart block	2 (2.5%)	0 (0%)	2 (4.3%)	1.4	0.5
- Tricuspid regurgitation	15 (18.8%)	0 (0%)	15 (31.9%)	12.9	< 0.001*
- Systemic hypertension	1 (1.3%)	0 (0%)	1 (2.1%)	0.7	1
- History of Thrombosis	1 (1.3%)	0 (0%)	1 (2.1%)	0.7	1

*Statistically significant at *p* ≤ 0.05

## Discussion

Monitoring iron overload in thalassemia syndromes is clinically critical for assessing the risk of existing complications and preventing future ones [5]. Excess iron can increase the production of free, unstable iron, which may, in turn, affect reactive oxygen species production and contribute to ongoing organ damage [14]. Although the quantitative assessment of serum ferritin broadly reflects body iron overload and is a more affordable and simpler tool [5], it may not fully reveal the total body iron burden and cardiac risk [15]. While ferritin has been found to independently predict a high risk of endocrine dysfunction, regardless of confounding factors [16], other studies indicate that serum ferritin is not a sufficient surrogate for cardiac iron evaluation [4]. This is because ferritin is an acute-phase reactant protein that can be non-specifically influenced by a wide range of inflammatory conditions or vitamin C deficiency [7, 16].

Moreover, other studies have shown that NTDT patients with serum ferritin levels between 300 ng/mL (the upper limit of normal) and 800 ng/mL consistently predict a liver iron concentration of 5 mg/g dry weight (dw) and remain at risk of iron-related morbidity [5]. Therefore, estimating liver iron concentration using non-invasive techniques remains critical in these cases to guide the assessment of the actual need for iron-chelating drugs [5]. Although the MRI scanner is a non-invasive technique for assessing liver iron concentrations, it carries clinical risks and restrictions and is contraindicated in various situations [17]. Hence, this case-control study of a thalassemia cohort is the first to evaluate the performance of circulating FABP4 as a predictor and diagnostic biomarker for thalassemia-associated cardiometabolic adverse events.

This cohort revealed that circulating FABP4 in thalassemia patients was significantly higher compared to non-obese control individuals. In line with earlier studies showing that circulating FABP4 is closely associated with obesity and metabolic syndrome [9, 18, 19], this finding highlights the significant role of macrophage in releasing FABP4 among the BMI-selected cohort. Although FABP4 is expressed at much higher levels in adipocytes compared to macrophages (approximately 10,000-fold) [8], its expression in macrophages overlaps significantly in function. It works with adipocytes to modulate the production of inflammatory cytokines and the accumulation of cholesterol ester, which can be induced by oxidized LDL and suppressed by statin therapy [9]. Interestingly, in an in vitro study, **Bosqut and his colleagues** (2018) demonstrated that the FABP4 inhibitor could reduce lipid-induced stress-associated inflammation in the endoplasmic reticulum, thus ameliorating lipid deposits and suppressing ROS [20].

Further, our results revealed a statistically significant positive correlation between serum FABP4 concentration and several laboratory parameters, including serum triglycerides (TGS), LDL, ALT, and ferritin. This suggests potential metaflammation in the thalassemia cohort. Additionally, patients with ferritin serum levels above 800 ug/L had statistically significant higher levels of serum FABP4. These findings are consistent with previous studies that demonstrated a significant positive association between elevated FABP4 levels and a cluster of metabolically driven low-grade chronic inflammation, including iron accumulation, type 2 diabetes mellitus, insulin resistance, atherogenic dyslipidemia, nonalcoholic steatohepatitis, and cardiovascular dysfunctions [13, 21–23].

Furthermore, earlier studies suggested that FABP4 may play a critical role in regulating cardiac depolarization and arrhythmias [4]. Although the Echo findings in this thalassemia cohort did not show a statistically significant correlation with circulating FABP4 concentration, the estimated FABP4 exclusionary cut-off value (> 2.30 ng/ml) was able to predict various cardiac dysfunctions in thalassemia patients, including congestive heart failure (CHF), arrhythmias, tricuspid regurgitation (TR), pulmonary and systemic hypertension, and a history of thrombosis. Consistently, many studies have identified CHF and fatal cardiac arrhythmias as the most serious cardiovascular problems in TDT patients [24–27]. The lower sensitivity of Echo in detecting all cardiac risks might explain the lack of significant differences observed in this thalassemia cohort.

In logistic regression analysis, FABP4 concentration was identified as a statistically significant risk factor for overall thalassemia-associated cardiometabolic events. The diagnostic ability of circulating FABP4 levels for predicting overall thalassemia-associated adverse metaflammation and subsequently cardiometabolic comorbidities was evaluated through ROC analysis, focusing on the area under the curves (AUC) for FABP4. Two cut-off points were determined: an exclusionary “rule out” cut-off for identifying cases free of cardiometabolic metflammation risk, and a confirmatory “rule in” cut-off for identifying cases at risk. The optimal cut-off value for ruling out cardiometabolic risk in this cohort was 2.30 ng/ml, which effectively discriminated between thalassemia patients with numerous cardiometabolic events, either clinically or sub-clinically evident through laboratory and Echo findings (thalassemia-associated metaflammation), and non-obese control individuals, with 90% sensitivity and 93.3% specificity. Conversely, the best confirmatory cut-off value for diagnosing and ruling in clinically evident cardiometabolic comorbidities was 2.58 ng/ml, with a sensitivity of 80% and a specificity of 60% for thalassemia-associated comorbidities.

Overall, this study highlights the importance of early clinical screening in thalassemia patients with FABP4 > 2.3 ng/ml to exclude cardiometabolic risks. Although the prevalence of thalassemia-associated cardiometabolic comorbidity in this cohort was 64%, limitations such as incomplete data on subclinical thyroid dysfunction and the unavailability of T2*MRI to assess total body iron overload due to limited resources must be acknowledged. Despite the modest sample size and the representation of a limited racial population, the statistical model was sufficiently robust to underscore that FABP4 is a key player in immunometabolic diseases. Further, FABP4 appears to be a promising risk stratification factor for thalassemia-associated cardiometabolic complications and a potential biomarker for predicting associated risks.

Interestingly, this study suggests that circulating FABP4 could be valuable for clinical monitoring of thalassemia-associated metaflammation and cardiometabolic comorbidities, potentially preventing future complications and optimizing iron-chelation therapy. Moreover, targeting FABP4 for therapeutic benefit may offer promise in reducing the burden of thalassemia-related comorbidities [13, 19]. A further prospective, multicentric, larger study comparing thalassemia-associated cardiometabolic abnormalities with the gold standard of iron overload assessment (T2*MRI) is recommended to confirm our findings and validate the diagnostic and prognostic performance of FABP4.

## Conclusion

Circulating FABP4 appears to be a potential risk factor for predicting the progression of cardiometabolic events associated with thalassemia-related adverse metaflammation. Therefore, it could serve as a valuable biomarker for disease monitoring and risk stratification in future prospective, multicentric, larger studies.

## Data Availability

No datasets were generated or analysed during the current study.
